# Predictive modeling of controlled drug release from polysaccharide-based systems using gradient boosting and metaheuristic optimization

**DOI:** 10.1038/s41598-026-48281-0

**Published:** 2026-04-22

**Authors:** Ahmed H. Albariqi, Abdullah Alsalhi, Meshal Alshamrani, Sameer Alshehri, Mahboubeh Pishnamazi

**Affiliations:** 1 Department of Pharmaceutics, College of Pharmacy, Jazan University, Jazan, 45142 Saudi Arabia; 2 Department of Pharmaceutics and Industrial Pharmacy, College of Pharmacy, Taif University, P.O. Box 11099, Taif, 21944 Saudi Arabia; 3 Institute of Research and Development, Duy Tan University, Da Nang, Vietnam; 4 School of Engineering & Technology, Duy Tan University, Da Nang, Vietnam

**Keywords:** Controlled drug release, Polysaccharide-based formulation, Metaheuristic optimization, Raman spectroscopy, Release kinetics modeling, Engineering, Mathematics and computing

## Abstract

Accurate prediction of drug release kinetics from polysaccharide-based delivery systems is essential for rational formulation design. In this study, a hybrid machine learning framework integrating Raman spectroscopy with formulation descriptors is developed to model drug release profiles across different polysaccharide matrices. A dataset comprising 155 experimental instances from 13 formulation groups is used, including 1,675 Raman spectral variables, categorical medium descriptors, and temporal information. Feature selection using F-statistics reduces the spectral space to 17 informative Raman peaks, which are combined with medium and time as model inputs. Extreme Gradient Boosting (XGB) and Light Gradient Boosting (LGB) models are optimized using Swarm-Assisted Bayesian Optimization (SABO) and Quantum-Inspired Optimization (QIO), forming four hybrid predictors (XGSO, XGQO, LGSO, and LGQO). The optimized hybrid models achieve superior predictive performance compared to single learners, with XGSO and LGSO yielding the lowest prediction errors (test RMSE = 0.065 and 0.077, respectively, and R^2^ = 0.961 and 0.939). SHapley Additive exPlanations (SHAP) reveal that Raman bands in the 940–990 cm^–1^ and 470–510 cm^–1^ regions, associated with glycosidic backbone vibrations of polysaccharides, exert the strongest influence on release prediction, together with time and medium effects. These results demonstrate that the proposed framework not only improves predictive accuracy but also captures chemically meaningful relationships between polymer structure and macroscopic drug release kinetics, supporting its potential for data-driven formulation optimization.

**Supplementary Information:**

The online version contains supplementary material available at 10.1038/s41598-026-48281-0.

## Introduction

As the primary sites of drug absorption are the stomach and small intestine, most commercially available medications are designed as oral solid dosage forms to enable effective release within these regions. Because oral administration is simpler, less intrusive, and less expensive than alternative administration methods, it is often preferred by patients and healthcare professionals^1,2^. Even during formulation immediate-release dosage are normally the preferredone, certain drugs work better if they are sent directly to the colon^3^. The colon may nonetheless provide pharmacokinetic, safety, and a smaller region for absorption instead of the tiny intestine and therapeutic advantages despite having a thicker epithelial mucus layer^4^. This is partially because, in comparison to the small intestine, it expresses less of the efflux transporter permeability-glycoprotein (P-gp) and the drug-metabolizing enzyme cytochrome P450 3A4 (CYP3A4)^5,6^. Colonic microbiota and nutrition-sensing receptors are among the local therapeutic targets that are related to systems disorders as well as can be accessed with the correction of the colon^7^. Targeted delivery to the proper location within the body can minimize side effects, dose needs, and variability in response, while simultaneously maximizing therapeutic efficacy^8,9^. Over the years, advances in the field of spectroscopic techniques, particularly Raman spectroscopy, have revolutionized the possibilities of drug formulations non-invasively being characterized as well as release behaviors at the molecular scale can be assessed^10,11^.

Abdalla et al.^12^ set the precedent to utilize Raman spectral data along with machine learning models to forecast the release of 5-aminosalicylic acid from polysaccharide coatings intended to promote colonic delivery. Their study identified the potential of Raman-based characteristics to retain polysaccharide structure and predict release kinetics with the potential to form a solid basis to fine-tune formulations. Similarly, Galata et al.^13^ compared Raman and near-infrared (NIR) imaging to forecast the dissolution profiles of extended-release tablets with artificial neural networks. The research indicated that both modalities had the potential to achieve high predictive accuracy, where NIR showed advantages to encourage the use of NIR in real-time industrial applications. Such papers suggested the increasing convergence between data acquisition of spectroscopic data and machine learning predictive modelling in the field of pharmaceutical research. Importantly, further marches have subsequently been derived in the use of Raman-based modes to realize real-time and in situ monitoring of drug delivery systems. Rath et al.^14^ availed themselves of defocused spatially-offset Raman spectroscopy (SORS) to non-invasively evaluate implant development alongside drug release kinetics, mapping the spectroscopic information onto solvent exchange along with burst release mechanisms. Sato et al.^15^ applied a probe-type Raman spectrometer in combination with partial least squares (PLS) analysis to quantify ciprofloxacin encapsulation in liposomal formulations, achieving less than 7% deviation from standard spectrophotometric techniques. These findings established Raman spectroscopy as an essential quality control tool for formulation monitoring. Moreover, Ganbold et al.^16,17^ demonstrated Raman spectroscopy’s capability to monitor intracellular release of anticancer drugs from nanoparticle carriers, revealing detailed kinetic mechanisms and validating the integration of multimodal spectroscopy for precise release characterization.

While previous research has demonstrated the potential of Raman spectroscopy and classical machine learning models, few studies have explored advanced ensemble-based and optimization-enhanced frameworks to model and forecast drug release behavior comprehensively^18–20^. Conventional models usually struggle with generalizability under varied formulation environments, especially when dealing with extensive spectral datasets containing hundreds of related variables. To suppress such limitations, this research presents a novel optimization ensemble learning framework for accurate drug release predictions. The model explicitly includes Light Gradient Boosting Regression (LGBR), Extreme Gradient Boosting Regression (XGBR), as well as an ensemble hybrid (LGBR–XGBR) tweaked with two modern metaheuristic optimization algorithms.

The proposed method leverages Raman spectral characteristics, categorical construct descriptors, and temporal information to establish a predictive scheme that can detect nonlinear high-dimensional dependencies prevalent in drug release systems. Statistical F-regression analysis is utilized to verify the significance of features, and SHAP (SHapley Additive exPlanations) interpretability is utilized to retrieve the most significant variables. Besides, the k-fold cross-validation ensures that the model generalizes strongly, and the Wilcoxon signed-rank test identifies the statistical significance of the proposed models over the standard practice. Finally, our study contributes to the field of intelligent pharmaceutical modeling by combining advanced gradient boosting techniques with modern optimization frameworks to enhance the accuracy of predicting drug release kinetics. The framework maximizes the accuracy of prediction with interpretability and real-use value towards future formulation development, quality control, as well as optimization of the process.

**Fig. 1:**
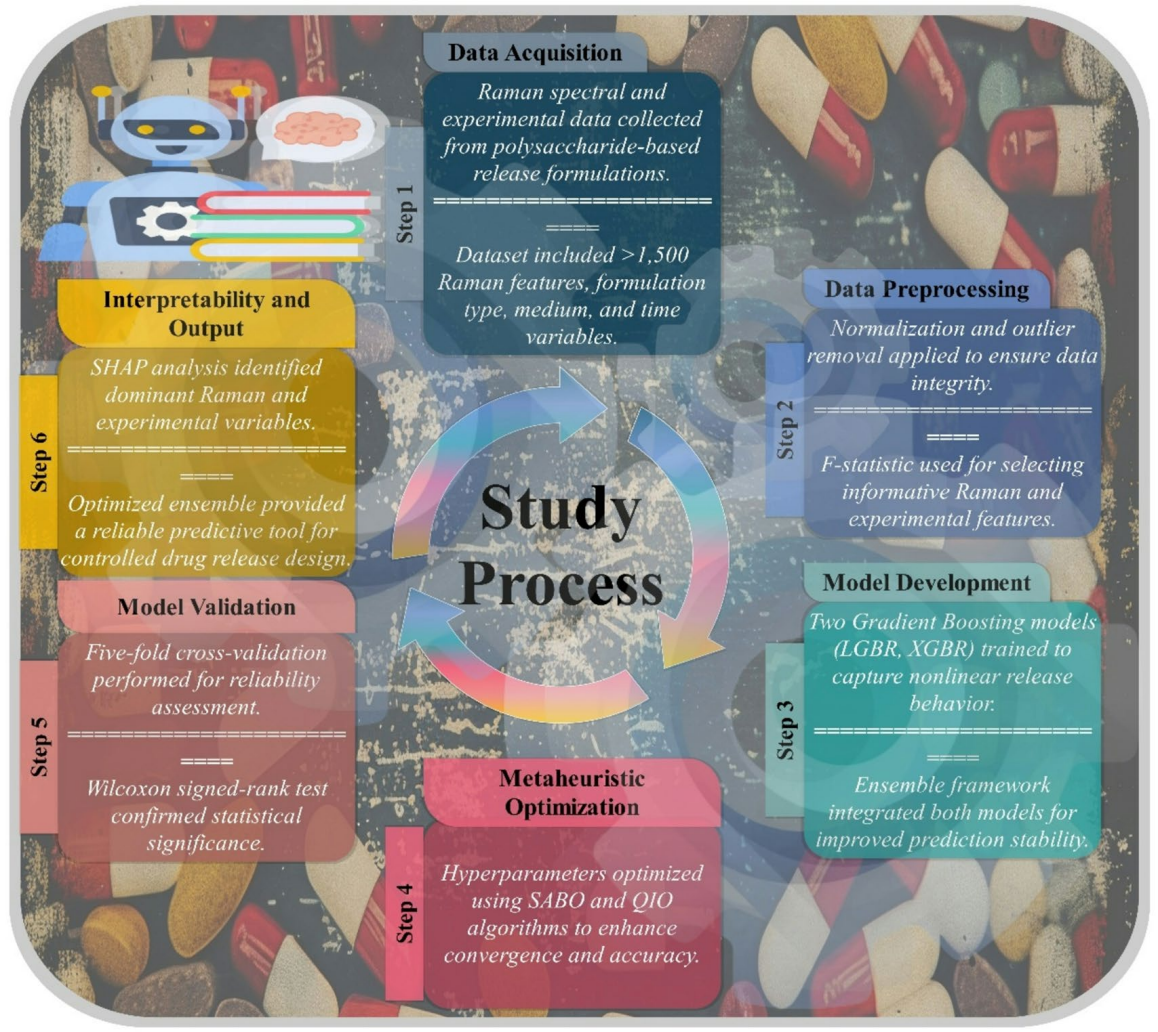
Study process and methodological framework.

Figure 1 illustrates the ordered work steps of the proposed optimization-improved ensemble learning framework to predict drug release behavior of polysaccharide-based formulations. The working is sequentially applied as follows: In data acquisition (Step 1), Raman spectra data as well as experimental data are obtained. Data preprocessing (Step 2) scale the data, eliminate the outlier to prevent the outlier to dominate the learning, and feature selection with the F-statistic to guarantee data quality. The model building (Step 3) entails learning two gradient boosting models (LGBR and XGBR) from the data, then combining the models as an ensemble to preserve nonlinear release kinetics. Metaheuristic optimization (Step 4) tunes the model hyperparameters guided by the SABO and QIO to facilitate accelerated convergence towards higher accuracy predictions. Model validation (Step 5) applies k-fold cross-validation and the Wilcoxon signed-rank test to establish the model’s robustness and statistical significance. Finally, interpretability and output analysis (Step 6) relies on SHAP values to identify the most impactful Raman and experimental features, thereby giving rise to an interpretable and credible tool to predict controlled drug release as well as formulate it.

## Materials and methodology

### Data gathering

**Fig. 2:**
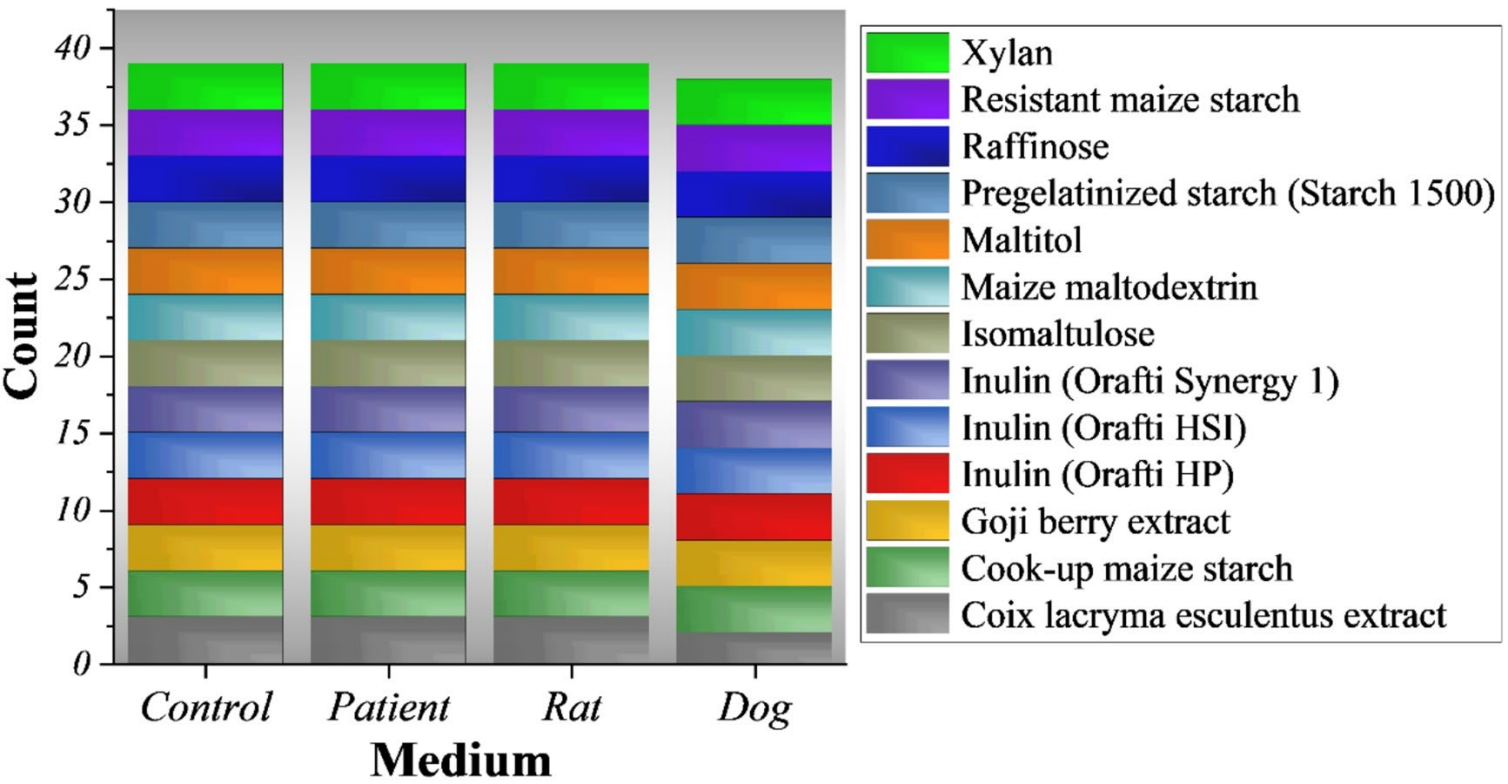
Distribution of polysaccharide coating types across different experimental media (Control, Patient, Rat, and Dog).

This section describes the full strategy for modeling and forecasting the target variable’s release behavior using a dataset of 155 data points, as previously published. The dataset may be retrieved via the GitHub link: https://github.com/y-babdalla/coating_release. A dataset with 155 data points is used in this investigation. The quantity of drug released at various times is represented by the objective variable “release,” which is intended to be predicted from a variety of inputs. More than 1,675 Raman spectral features in the dataset offer comprehensive details about the vibrational properties of the data points. Additionally, it has a numerical variable called “time” that represents the duration of the experiment. Moreover, two category variables, “polysaccharide name” and “medium”, describe the composition and environmental context that could affect the outcome variable. In the modeling process, four distinct media were considered: Control, Patient, Rat, and Dog (Fig. 2). Each colored segment represents the count of a specific polysaccharide type used in the dataset, including Xylan, Resistant maize starch, Raffinose, Pregelatinized starch, Maltitol, Maize maltodextrin, Isomaltulose, various Inulin types (Orafti Synergy 1, Orafti HSI, Orafti HP), Goji berry extract, Cook-up maize starch, and Coix lacryma esculentus extract. The stacked bar representation illustrates the balanced presence of polysaccharide formulations across all four media, ensuring diversity and representativeness of experimental conditions for predictive modeling of drug release behavior.

### Data preprocessing and dimensionality control

The dataset consisted of 155 observations described by more than 1,500 Raman spectral variables, in addition to categorical descriptors (polysaccharide type and medium) and a temporal variable (time). Such a configuration presents a high-dimensional learning problem in which the number of predictors greatly exceeds the number of samples, potentially leading to overfitting and instability, commonly referred to as the curse of dimensionality. To address this issue, a dimensionality reduction strategy based on statistical feature selection was implemented prior to model training. All continuous variables were first normalized to ensure numerical comparability and to prevent scale dominance. Outliers were subsequently identified and removed using statistical thresholds to minimize the influence of abnormal values.

Feature selection was then conducted using an F-statistic ranking approach to identify Raman spectral features most strongly associated with the release response variable. F-statistics reduced the original 1,675 Raman spectral variables to 17 informative Raman features. Together with the categorical variable medium and the temporal variable time, a total of 19 input variables were used for model training, while release was treated as the target output. To avoid selection bias and information leakage, feature selection was embedded within the cross-validation framework. For each fold of the k-fold procedure, the F-statistic ranking was computed exclusively on the training subset, and only the selected features were transferred to the corresponding validation subset. This ensured that no information from validation samples contributed to feature selection or model calibration, thereby preserving the independence of performance estimation. Although an independent external dataset was not available for hold-out testing, the use of fold-wise feature selection combined with repeated cross-validation provides a conservative and statistically sound approximation of out-of-sample performance. This strategy minimizes optimistic bias and supports robust learning under limited sample conditions typical of experimental spectroscopic datasets.

#### Feature selection analyses

As mentioned, feature selection was done using f-statistics. Thus, out of 1675 entries, 20 entries remained. Table 1 shows the statistical characteristics of these variables, where P stands for polysaccharide name and the index is the experimental design included 13 treatment groups: a control group (maize maltodextrin), six carbohydrate or fiber-based prebiotic groups (inulin-SYN, inulin-HSI, inulin-HP, xylan, resistant starch, and raffinose), two comparator carbohydrate groups (maltitol and isomaltulose), two starch control groups (pregelatinized starch and cook-up maize starch), and two bioactive extract groups (goji berry extract and Coix lacryma-esculentus extract).

To ensure reproducibility and transparency, the supplementary Excel file has been provided to include the original dataset and dataset after feature selection. Feature selection was conducted using an F-statistic ranking approach to identify Raman spectral variables most strongly associated with the drug release response. From the original set of 1,675 Raman wavenumbers, 17 Raman features were retained: 989.754883, 941.882813, 940.738281, 939.592773, 938.447266, 902.81543, 901.662109, 900.508789, 510.868164, 483.564453, 482.319336, 481.075195, 479.830078, 478.584961, 477.339844, 476.094727, and 474.848633 cm^–1^. In addition to these spectral predictors, the categorical variable medium and the temporal variable time were preserved as auxiliary inputs, while release was defined as the target variable. The index column was retained only as a sample identifier and was excluded from the modeling process.The selected Raman features correspond to discrete spectral peaks rather than contiguous spectral bands, indicating that the feature selection procedure isolated chemically informative vibrational modes instead of broad spectral regions. These peaks are mainly concentrated in the 940–990 cm^–1^ and 470–510 cm^–1^ ranges, which are associated with polysaccharide backbone vibrations and hydration-sensitive modes. To ensure reproducibility, the supplementary Excel file has been updated to include the dataset after feature selection, explicitly reporting the retained Raman shifts together with the corresponding experimental and categorical variables. This enables independent reconstruction of the feature space and supports the chemical interpretation of the SHAP-based importance analysis.

**Table 1:** Input variables consideration.

Distribution	Variable	Indicator					
		Lower bound			Upper bound		
Uniform	Index	1			44		
	Medium	1			4		
		Max.	Min.	Avg.	St. Dev.	Skewness	Median
Normal	P (989.75)	383129.0	989.8	55740.1	96819.2	2.929	19854.5
	P (941.88)	409277.9	941.9	61936.9	102978.7	2.908	32083.2
	P (940.74)	411085.0	940.7	62382.8	103408.5	2.905	33804.2
	P (939.59)	411626.5	939.6	62568.1	103483.5	2.908	34271.1
	P (938.45)	411210.8	938.4	62438.7	103377.2	2.911	34860.7
	P (902.82)	400460.1	902.8	60575.8	100904.1	2.891	20848.8
	P (901.66)	399616.5	901.7	60223.6	100824.6	2.885	20247.0
	P (900.51)	400893.2	900.5	59739.7	101310.3	2.890	19903.3
	P (510.87)	496136.0	510.9	78360.3	123457.5	2.935	32212.3
	P (483.56)	523729.2	483.6	87165.7	130421.5	2.825	56103.1
	P (482.32)	524027.1	482.3	88576.9	130376.1	2.803	57888.4
	P (481.08)	526810.1	481.1	89927.3	130980.2	2.788	58091.7
	P (479.83)	526060.9	479.8	90920.4	130646.3	2.774	58273.9
	P (478.58)	524704.0	478.6	91224.4	130232.2	2.767	56999.1
	P (477.34)	522953.8	477.3	91161.9	129736.9	2.766	54723.1
	P (476.09)	520889.9	476.1	90338.3	129179.6	2.780	52286.8
	P (474.85)	518278.1	474.8	88709.2	128525.0	2.807	49482.8
	time	24.0	2.0	11.4	9.3	0.5	8.0
	release	1.0	0.0	0.2	0.3	1.3	0.1

#### K-fold cross validation

Table 2 presents the performance of the developed models using a 5-fold cross-validation approach. The results show that both the XGB and LGB models achieved consistent and reliable performance across all folds, indicating strong model stability. For the RMSE indicator, the XGB model yielded values ranging from 0.075 to 0.096, with an average of 0.083 ± 0.009, while the LGB model showed slightly higher variability, with RMSE values between 0.081 and 0.107 (average 0.092 ± 0.010). Lower RMSE values reflect smaller prediction errors, suggesting that the XGB model provided more accurate estimations of the target variable. Similarly, the R^2^ values confirm this trend. The XGB model achieved an average R^2^ of 0.927 ± 0.009, slightly outperforming LGB (0.909 ± 0.009). The high R^2^ values (> 0.90) for both models demonstrate that a substantial proportion of the variance in the data was successfully explained.

**Table 2:** The result of developed K-Fold.

Models	Indicator	Number of K-Fold				
		K1	K2	K3	K4	K5
XGB	RMSE	0.096	0.075	0.078	0.089	0.078
LGB		0.096	0.086	0.091	0.107	0.081
XGB	R^2^	0.912	0.937	0.932	0.922	0.931
LGB		0.908	0.914	0.912	0.893	0.918

#### Computational validation strategy

The modeling framework was developed and validated using a benchmark dataset obtained from a previously published experimental investigation, as detailed in section “Data gathering”. The dataset consists of 155 experimentally measured drug release profiles derived from 13 polysaccharide-based formulation groups, evaluated under four dissolution media (Control, Patient, Rat, and Dog), alongside corresponding Raman spectroscopic measurements. The original experimental procedures included in vitro dissolution testing and Raman spectral acquisition under controlled laboratory conditions. These experimentally validated data provide a reliable basis for assessing predictive modeling strategies without the need for redundant experimental replication.

To ensure rigorous validation of the proposed predictive framework, a comprehensive computational evaluation methodology was employed. A stratified 5-fold cross-validation scheme was implemented to assess model generalization across different formulation compositions and dissolution environments. Feature selection using the F-statistic ranking approach was embedded within each training fold to prevent information leakage and ensure unbiased performance estimation. Model performance was quantitatively evaluated using multiple complementary statistical metrics, including root mean square error (RMSE), coefficient of determination (R^2^), mean absolute error (MAE), scatter index (SI), and Theil’s inequality coefficient (TIC). These metrics jointly characterize prediction accuracy, stability, and robustness.

Additionally, non-parametric statistical significance testing using the Wilcoxon signed-rank test was conducted to evaluate the consistency between predicted and experimentally measured drug release values. The absence of statistically significant differences (*p* > 0.05) confirms that the predictive models reliably reproduce the experimental trends reported in the literature dataset. This validation framework ensures that model performance reflects true predictive capability rather than dataset memorization.

### Machine learning models

The study utilized two state-of-the-art ensemble-based machine learning models, LGB and XGBR, to precisely model and predict the target variable. They are tree-based gradient boosting frameworks that work to detect complex nonlinear interactions and improve predictions by adjusting errors and learning feature interaction learning with each other.

#### Light Gradient Boosting (LGB)

LGB is a recently developed and highly efficient framework of gradient boosting that is notorious for being capable of processing high volumes of data, being efficient in terms of computer operation, and predicting with high accuracy. The boosting procedure reduces the residual error in a systematic manner to construct an ensemble of decision trees. This combines a strong composite from numerous weak learners. The fact that LGB creates decision trees based on histograms which divide continuous attributes into bins is among its most significant innovations. This approach significantly reduces processing time and memory usage, and it also includes an inbuilt regularization mechanism that guards against overfitting^21–23^. Additionally, LGB eliminates pre-sorted features, making it capable of parallel processing and highly effective for high-dimensional, large-scale datasets. Its leaf-wise tree growth strategy, in which the algorithm decides to enlarge the leaf with the highest information gain rather than descending trees level by level, is arguably its most noticeable distinction. This architecture improves model accuracy by enabling LGB to identify complex feature interactions with fewer iterations. The algorithm also includes regularization terms and depth limitations to help achieve the ideal balance between model flexibility and generalization, preventing overfitting that can happen when the trees are deep.

**Fig. 3:**
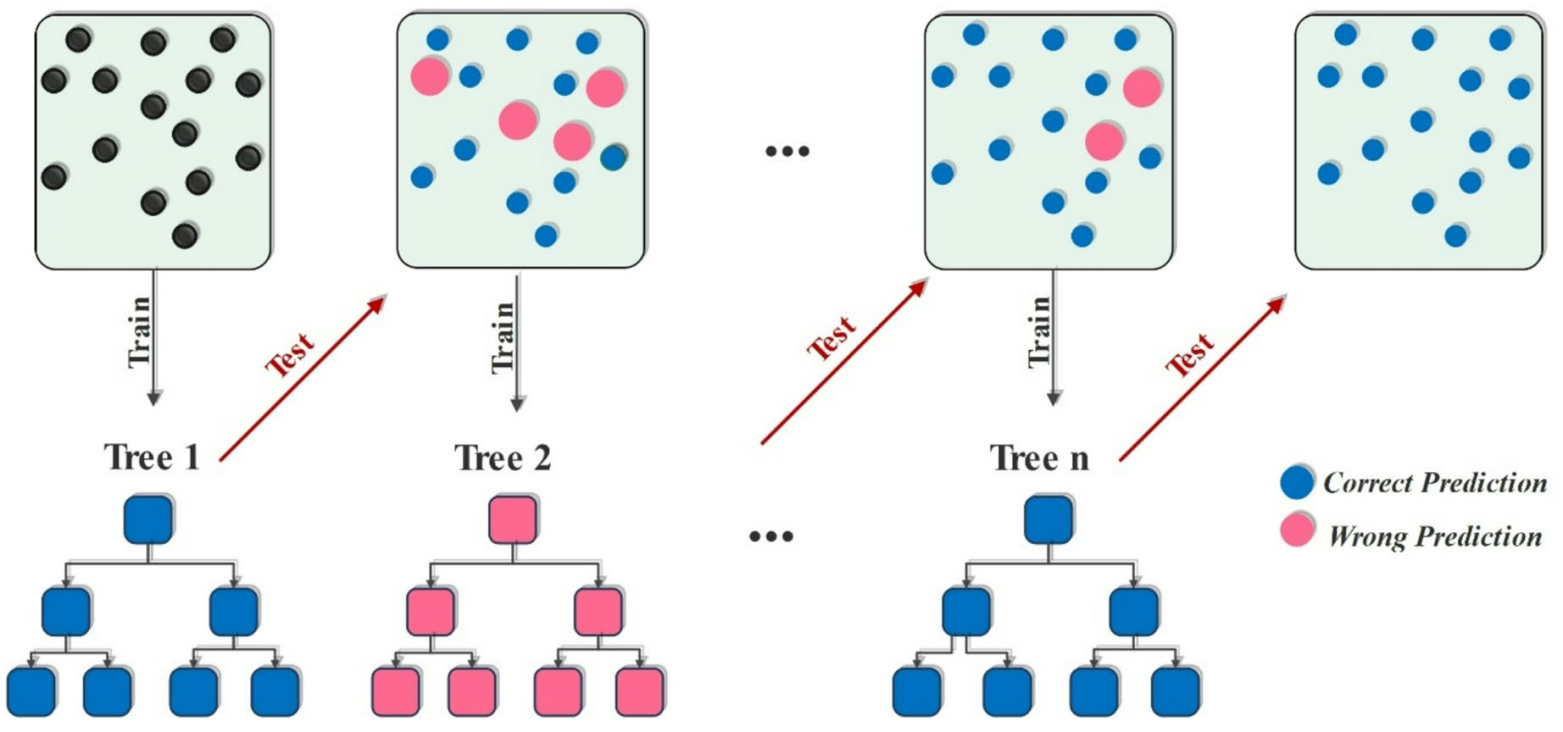
Schematic illustration of the LGB model architecture and training process.

Figure 3 illustrates the organization of the LGB models that were employed in the research. The figure depicts the sequential learning method of the boosting framework, whereby each decision tree is utilized to fix the prediction errors of the previous one. Incorrectly predicted data points (pink) during the iterative operations are assigned more weights, and hence the new trees are “guided” to concentrate on these samples that are more difficult to predict. Thus, by combining many weak learners (trees) the model becomes a strong predictive one with improved accuracy and generalization capability. Nodes and samples that are shown in blue represent correct predictions whereas pink nodes correspond to misclassifications or residual errors that are dealt with in the next boosting rounds.

#### Extreme Gradient Boosting Regression (XGBR)

An enhanced and distributed variant of the gradient boosting framework, XGBR is intended to be highly effective and robust. By creating a series of decision trees, each of which corrects the errors of the one before it, it improves predictions^24^. A regularized objective function with a complexity penalty and a loss term is used by XGBR. This enhances the model’s capacity for generalization and keeps it from overfitting. Its use of second-order gradient statistics accelerates convergence and stabilizes learning, and its adaptive binary splitting algorithm ensures that every tree split receives the most information^25^. XGBR is also strong against outliers and noise since it has built-in regularization and shrinking methods. Overall, XGBR is a good framework for complicated regression problems that include nonlinear and multivariate relationships because it has excellent prediction accuracy, fast processing, and great interpretability.

#### Justifications of gradient boostung models

The choice of LGB and XGBR models is mainly driven by their good ability to cope with high-dimensional, feature-enormous datasets and nonlinear structure to capture complex nonlinear associations between predictors. Here, the dataset is comprised of over 1,500 Raman spectral features, categorical variables that represent the type of polysaccharide and medium, along with a continuous variable representing time, with the aim to predict the drug release rate. The nature of such a dataset is high heterogeneity, inter-feature correlation, as well as nonlinear interactions that are hard to capture with traditional regression or flat ‏learning schemes. Gradient boosting models are best suited to such data ‏because they successively improve predictions by focusing on misrepresented ‏observations, allowing the model to detect subtle variations within the Raman spectra that ‏affect drug diffusion as well as release kinetics. The reason why LGB is selected is that it has histogram-based feature handling along with a leaf-wise tree growth strategy that reduces ‏overfitting with efficient computational cost to detect fine-grained spectral features. Its scalability ‏and ability to handle large volumes of highly correlated inputs are best suited for Raman ‏based prediction tasks.

Similar to this, XGBR was selected due to its robust regularization mechanisms and use of both first- and second-order gradient information, which enhance feature contribution interpretability and stabilize the optimization process. The fact that it is resistant to outliers and noise is very useful, in particular, when experimental data are modeled that are the result of the biological or chemical systems, in which there is always some variability. Hence, by combining LGB and XGBR, a robust framework is achieved which is capable of learning complex feature interactions from the spectral, categorical, and temporal domains and thus can make accurate and generalizable predictions of drug release behavior. These models offer an interpretable and computationally efficient basis for the creation of optimized predictive tools in pharmaceutical release modeling.

### Optimization algorithms

Optimization algorithms are crucial to improve the effectiveness, precision, and robustness of machine learning models through adjustment of their parameters and generalization improvement. In this study, the hyperparameters of the presented models were adjusted using SABO and QIO, two excellent metaheuristic optimization algorithms. The two algorithms are designed to strike a balance between exploitation (fine-tuning promising regions to converge to the best solution) and exploration (exploring the global region to search for varied candidate solutions). When compared to traditional optimization techniques, their integration guarantees that the models achieve faster convergence, higher prediction accuracy, and superior stability.

#### Subtraction-Average-Based Optimizer (SABO)

**Fig. 4:**
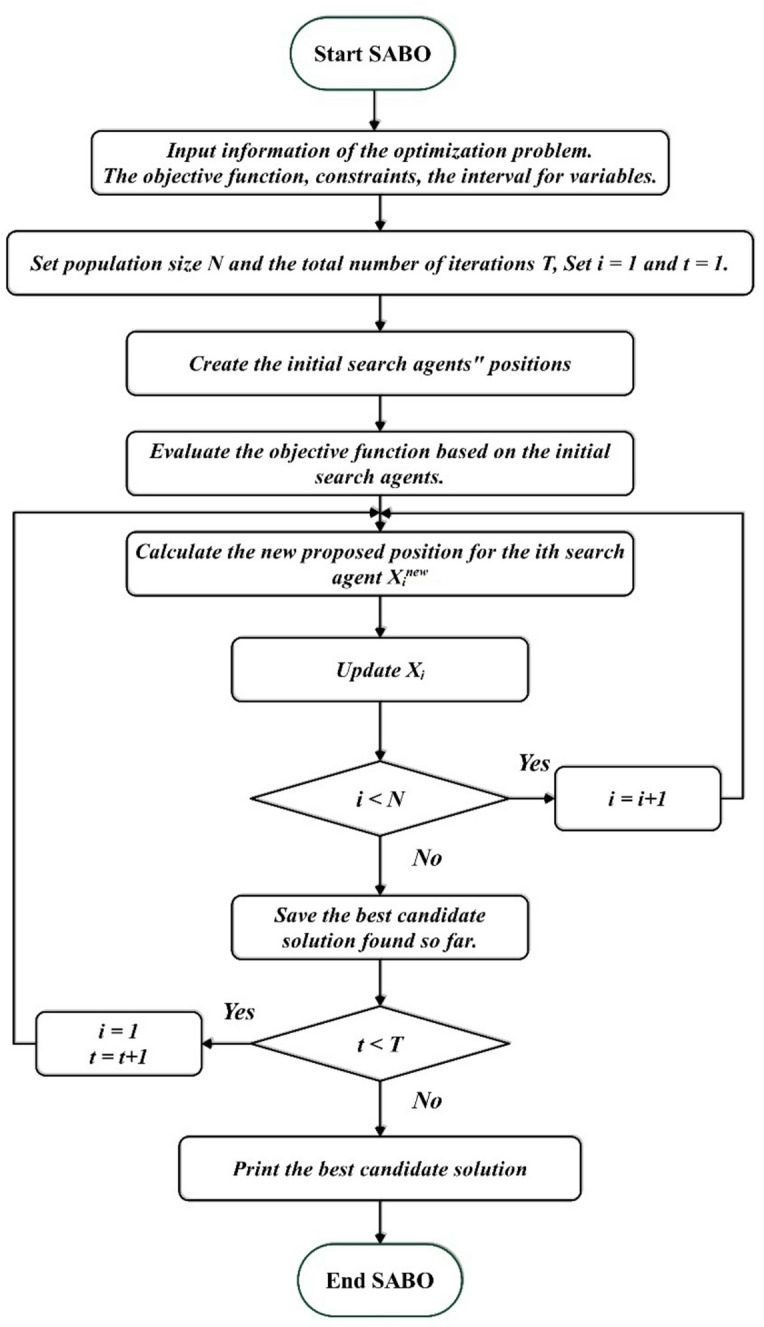
Flowchart of the Subtraction-Average-Based Optimizer (SABO).

The SABO is a new metaheuristic algorithm that makes searches more efficient by combining averaging and differential subtraction method^26^. In SABO, every search entity represents a solution in a multidimensional search space. In order to preserve population diversity, the entities are first dispersed at random. The algorithm then evaluates each entity’s performance and adjusts its locations by calculating the mean of its relative differences—a process known as the subtraction-average process. With the aid of this cooperative learning approach, SABO is able to conduct a broad initial exploration and then progressively employ the most promising areas as the iterations progress^27,28^. While the averaging operation directs convergence toward the global optimum, the subtraction operation encourages diversity and aids in escaping local minima. SABO makes sure that only better candidates are kept by regularly comparing new and existing solutions, which promotes steady convergence and progress. SABO is especially useful for complicated optimization tasks like hyperparameter tuning in ensemble learning models because of its versatility and well-balanced design. The SABO algorithm flowchart, shown in Fig. 4, starts with setting the optimization parameters, such as the population size, number of iterations, and variable bounds. The objective function is used to evaluate the search agents’ fitness after their positions are randomly generated within the search space.

#### Quadratic Interpolation Optimization (QIO)

The generalized quadratic interpolation procedure provided the motive behind the QIO algorithm, a modern metaheuristic. It predicts the fitness landscape as well as the future best routes of search with the aid of adaptive quadratic modeling^29,30^. Exploration, that sprawls the population to diversify the population as well as prevent early converge, and exploitation, that converges the research towards the best-performing solutions, are the two main phases of the operation of QIO. To generate new candidate solutions during the exploration step, QIO uses random sampling as well as interpolation, that allow the algorithm to analyze a high number of potential optima. The use of quadratic estimation makes the convergence faster with numerical stability maintained during exploitation, focusing on the region around the best solution currently at the best solution^31,32^. QIO can address nonlinear and multimodal issues efficiently due to the harmonic coexistence between exploration and refinement. QIO is best suited to hyperparameter optimization of machine learning due to the predictive nature as well as the adaptive learning strategy. It can be a valuable tool in the improvement of model generalization as well as predictive ability due to the high convergency speed, preciseness, as well as noising resistance.

#### Justifications of optimization selection

Owing to their proven performance in solving complex, nonlinear, and high-dimensional optimization issues, the QIO and SABO were selected. The algorithms possess superior global search potential, are more adaptable, and more insensitive to local minima compared to traditional gradient-based or exhaustive search procedures. SABO was selected due to its cooperation learning mechanism that applies the subtract-average process to achieve the equilibrium between exploration and exploitation as well as effective population diversity stability during convergence. By employing quadratic interpolation to approximate the fitness landscape, QIO, however, facilitates fast and precise convergence to the global optimum. An effective optimization framework that reduces model precision improvement, computational complexity, and assures uniform performance with different data distribution is afforded by the collaborative nature between QIO’s local refinement as well as SABO’s global exploration.

### Uncertainty estimation

Estimating uncertainty is a crucial aspect of verifying the strength and reliability of predictive models, particularly in the case of working with experimental data that is inherently variable, such as drug release activity from systems based on polysaccharides. This analysis seeks to investigate the stability of the best-performing gradient booster models under different conditions of data and to establish the level of confidence that can be placed in model predictions. The statistical scatter of prediction errors along with the uniformity of the model performance metrics across a number of validation folds were examined in this case to establish a measure of uncertainty. The use of the k-fold cross-validation enabled a credible assessment to be made of how prediction performance depends on different splits of the data into trains and tests, highlighting the variance of model accuracy. Good stability of the model along with limited predictive uncertainty are evident from the low variance of metrics such as RMSE and R2 across the folds. Moreover, the ensemble nature of the LGB and XGBR models inherently diminishes uncertainty since multiple weak learners cooperatively collaborate to dampen random fluctuations induced due to noise or outlier influences. By tuning the hyperparameters towards configurations that proceed to consistently enhance model generalizability, the SABO and QIO optimization procedures are included, augmenting robustness even further. Overall, the uncertainty analysis supports the suggested optimized models’ applicability for accurate drug release dynamics prediction under a variety of experimental settings by confirming that they not only achieve high predictive accuracy but also retain robustness and consistency across various data partitions.

### Methodological rationale in relation to research findings

The methodological workflow adopted in this study was designed to align directly with the objective of identifying chemically meaningful predictors of drug release behavior. The use of Raman spectroscopy provided molecular-level information on the polysaccharide matrices, while the inclusion of medium and time variables enabled incorporation of environmental and kinetic effects. Feature selection was applied not only to reduce dimensionality but also to isolate Raman bands associated with polysaccharide backbone vibrations, which were later confirmed by the SHAP analysis to be among the most influential predictors. The choice of gradient boosting models was motivated by their capacity to capture nonlinear interactions between spectral features and formulation variables, which is consistent with the observed improvement in predictive accuracy for hybrid models. Furthermore, the use of SHAP-based interpretability ensured that the resulting predictions could be traced back to specific Raman shifts and formulation conditions, thereby enabling chemical interpretation of the learned relationships. This integrated strategy allows the framework to move beyond empirical curve fitting and toward mechanistically informed prediction of drug release kinetics.

### Performance evaluation metrics

The root mean square error (RMSE), coefficient of determination (R^2^), mean absolute error (MAE), scatter index (SI), and Theil’s inequality coefficient (TIC) are five supportive statistical indices that were utilized to thoroughly analyze the predictive reliability of the proposed models. Using both spectral and experimental features, the metrics evaluate the models’ accuracy, constancy, and strength in predicting the drug release rate from polysaccharide-based systems. Performance assessment must take into consideration both absolute prediction accuracy as well as relative error sensitivity since the dataset comprises over 1,500 Raman spectral descriptors, categorical variables (polysaccharide type and release medium), as well as the continuous time variable.

As such, the RMSE and MAE quantify the extent of variation between the release values observed and release values predicted, reflecting the performance of the models in transferring feature data such as temporal trends and spectral intensity into accurate release predictions. Lower values of these indices reflect higher conformity to experimental data as well as better model calibration. The R^2^ statistic estimates the extent to which the variance in the experimental release data can be described from the model predictions. High R^2^ values reflect the success of the QIO and SABO-enhanced LGB and XGBR models in extracting the nonlinear and multivariate associations between medium composition, release kinetics, and Raman features. The SI, defined as the ratio of RMSE to the mean observed release value, offers a normalized quantity representing the prediction error that is insensitive to the scale of experimental measurerecording, enabling the comparison of performance between media as well as across time intervals. The TIC further assesses the overall forecasting efficiency by evaluating the relative magnitude of prediction errors to the combined energy of predicted and actual data. A smaller TIC denotes superior model alignment with real-world release behavior and minimal systematic bias. Collectively, these evaluation metrics provide a multidimensional framework for assessing predictive performance, ensuring that the developed models demonstrate high accuracy, stability, and generalization capability when applied to complex experimental datasets describing drug release kinetics under varying conditions.

1 $$RMSE=\sqrt {\frac{1}{n}\mathop \sum \limits_{{i=1}}^{n} {{\left( {{y_i} - \widehat {{{y_i}}}} \right)}^2}}$$

2 $${R^2}=1 - \frac{{\mathop \sum \nolimits_{{i=1}}^{n} {{\left( {{y_i} - \widehat {{{y_i}}}} \right)}^2}}}{{\mathop \sum \nolimits_{{i=1}}^{n} {{\left( {{y_i} - \bar {y}} \right)}^2}}}$$

3 $$MAE=\frac{1}{n}\mathop \sum \limits_{{i=1}}^{n} \left| {{y_i} - \widehat {{{y_i}}}} \right|$$

4 $$SI=\frac{{RMSE}}{{\bar {y}}}$$

5 $$TIC=\frac{{\sqrt {\frac{1}{n}\mathop \sum \nolimits_{{i=1}}^{n} {{\left( {{y_i} - \widehat {{{y_i}}}} \right)}^2}} }}{{\sqrt {\frac{1}{n}\mathop \sum \nolimits_{{i=1}}^{n} y_{i}^{2}} +\sqrt {\frac{1}{n}\mathop \sum \nolimits_{{i=1}}^{n} \widehat {{{y_i}}}_{i}^{2}} }}$$

## Results and discussion

### Optimization performance and convergence behavior

Table 3 summarizes the optimized hyperparameters and convergence behavior obtained using the SABO and QIO for the XGB and LGB models. Both optimizers achieved rapid and stable convergence while adapting effectively to the nonlinear and high-dimensional nature of the Raman-based dataset. For the XGB model, SABO reached convergence within 200 iterations, generating a compact configuration (N estimators = 217, max depth = 335, learning rate = 0.369) with strong regularization (Reg α = 0.999). This setting reflects efficient global exploration and a balanced bias–variance trade-off. Conversely, the QIO-optimized XGB adopted deeper trees and a lower learning rate (max depth = 853; learning rate = 0.0399), emphasizing fine local adjustment and enhanced precision in capturing subtle nonlinear relations among spectral features. For the LGB model, SABO again converged at 200 iterations, yielding an aggressive learning configuration (learning rate = 0.481; N estimators = 966) suitable for rapid error reduction. The QIO-based LGB, however, preferred a more conservative structure (learning rate = 0.4035; N estimators = 266) and finer bin resolution (max bin = 726 829), indicating a focus on detailed feature partitioning and smoother gradient transitions. Overall, SABO exhibited stronger global exploration and early stabilization, whereas QIO provided refined local optimization. Their complementary search behaviors produced tuned models with superior convergence stability and predictive readiness, aligning model complexity with the intrinsic variability of polysaccharide-based drug release data.

**Table 3:** Optimized hyperparameters and convergence iteration numbers, presenting the model tuning details and optimization performance.

Models	Optimizers	Convergence (Iterations)	Optimized hyperparameters	
XGB	Subtraction-Average- Based Optimizer	200	$N~estimators$	217
			$Max~depth$	335
			$Learning~rate$	0.369
			$Colsample~bytree$	0.999
			$subsample$	0.951
			$Reg~alpha$	0.999
	Quadratic Interpolation Optimization		$Reg~lambda$	0.352
			$N~estimators$	973
			$Max~depth$	853
			$Learning~rate$	0.039953
			$Colsample~bytree$	0.901034
			$subsample$	0.942458
			$Reg~alpha$	0.888763
LGB	Subtraction-Average- Based Optimizer	200	$Num~leaves$	285
			$Max~depth$	500
			$Learning~rate$	0.481
			$N~estimators$	966
			$Max~bin$	282,000
	Quadratic Interpolation Optimization		$Num~leaves$	688
			$Max~depth$	692
			$Learning~rate$	0.403512
			$N~estimators$	266
			$Max~bin$	726,829

### Predictive performance, error distribution, and learning dynamics

**Fig. 5:**
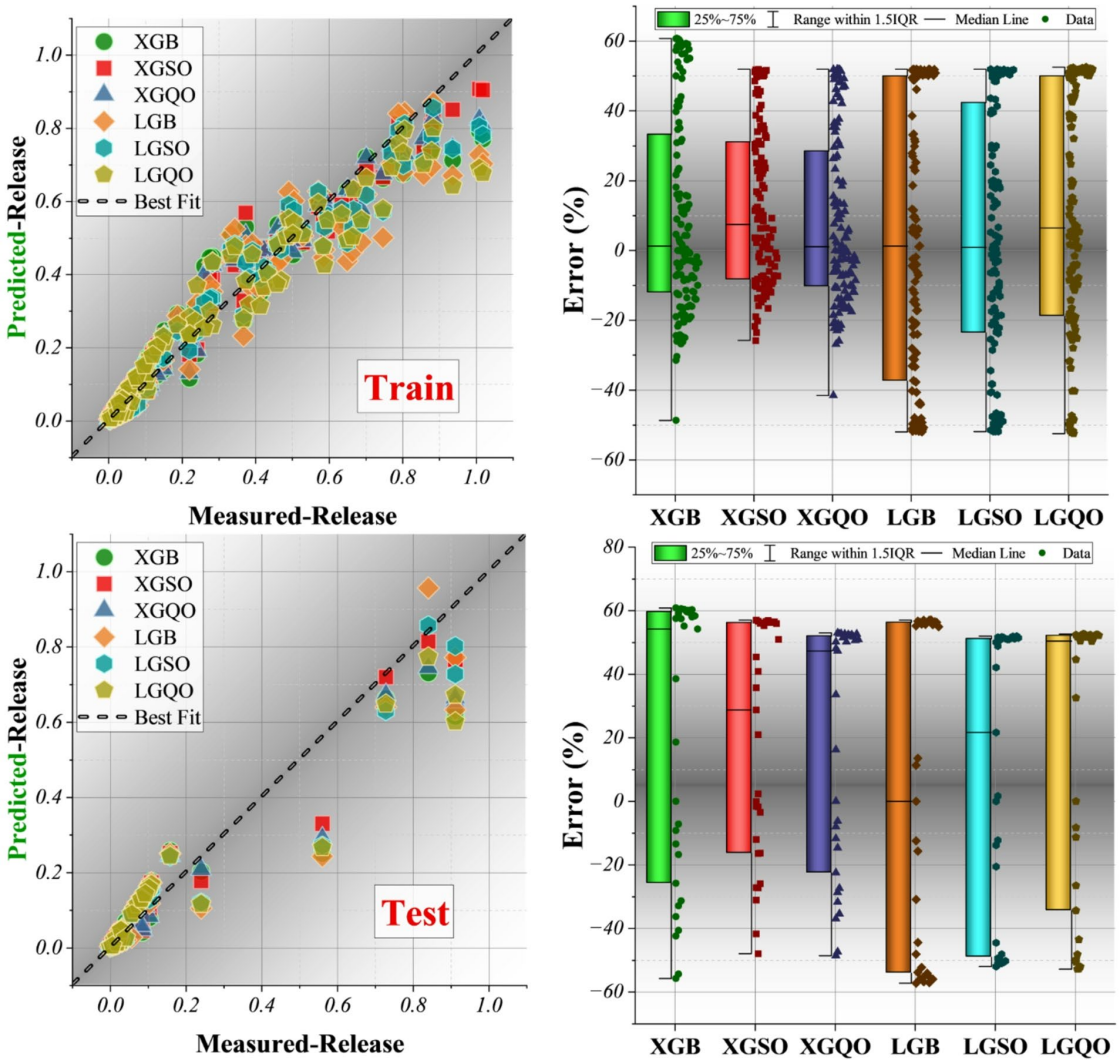
Scatter plot with adjacent half box plot illustrating the distribution and spread of prediction errors, highlighting model accuracy and error variability.

**Fig. 6:**
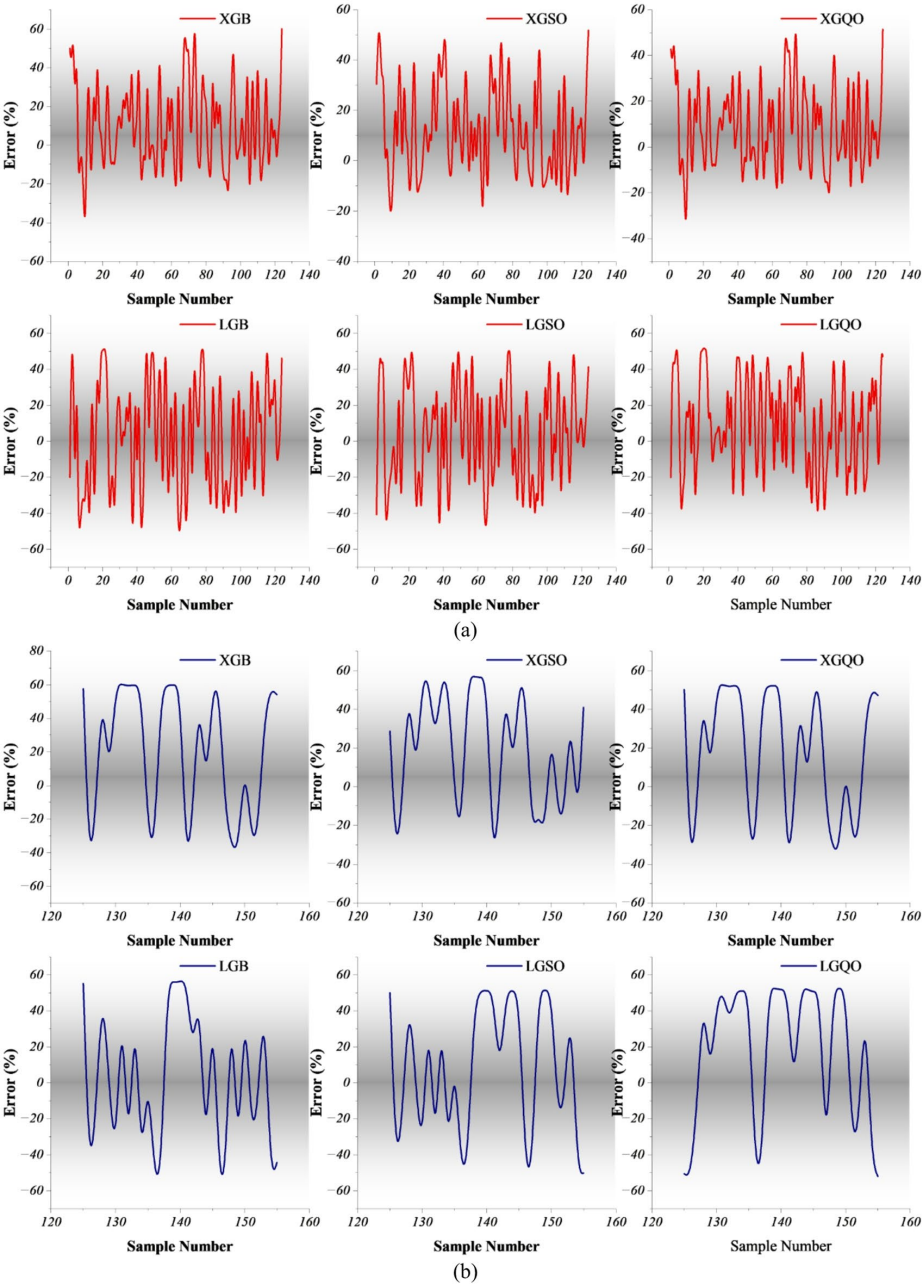
Line plot for error variation across iterations: (a) training phase and (b) testing phase, demonstrating the model’s learning behavior and generalization performance.

The predictive outcomes of the developed ensemble and hybrid frameworks reveal the model’s intrinsic ability to capture complex, nonlinear dependencies within the Raman spectral, compositional, and temporal domains governing drug-release kinetics. The statistical performance indicators summarized in Table 4, supported by the visual analyses in Figs. 5 and 6, collectively highlight the distinctive predictive behavior, feature-response alignment, and learning stability of the optimized architectures.

As presented in Table 4, all models achieved high predictive precision (R^2^ > 0.89), confirming their capability to map spectral signatures and experimental variables to measured release behavior. The hybrid models, particularly XGSO and XGQO, exhibited the lowest RMSE and MAE values in both training and testing phases (RMSE = 0.046–0.065), reflecting the enhanced parameter synergy induced by metaheuristic-guided optimization. The integration of SABO and QIO refined the learning rate and tree depth, allowing the models to isolate feature clusters representing Raman bands associated with polysaccharide backbone vibrations (~ 940–990 cm^–1^) and moisture-related interactions below 500 cm^–1^. This feature–response coupling explains the model’s capacity to generalize across varying release media while preserving molecular specificity.

In addition to the proposed SABO and QIO based optimization strategies, conventional hyperparameter tuning approaches including Random Search (RS) and Bayesian Optimization (BO) were evaluated as benchmark optimizers. Both RS and BO improved the predictive accuracy of the base XGB and LGB models relative to their non-optimized counterparts, confirming the importance of hyperparameter calibration for spectroscopic learning tasks. However, the SABO- and QIO-optimized hybrid models consistently outperformed the RS- and BO-optimized variants across all evaluation metrics. In the testing phase, XGB-BO achieved an RMSE of 0.079 and R^2^ of 0.946, whereas the SABO-optimized hybrid model (XGSO) reduced the RMSE further to 0.065 and increased R^2^ to 0.961. This corresponds to an additional error reduction of approximately 8–12% beyond Bayesian Optimization. These gains highlight the advantage of the metaheuristic search behavior of SABO and QIO in navigating the rugged hyperparameter landscape induced by high-dimensional Raman features and ensemble tree interactions. Unlike BO, which relies on surrogate modeling and may suffer from local exploitation bias in irregular objective spaces, SABO and QIO maintain population diversity and adaptive interpolation, enabling more robust convergence toward globally optimal configurations.

**Table 4:** Performance metrics of the models, assessing their predictive accuracy and effectiveness using key statistical indicators.

Process	Category	Models	RMSE	*R* ^2^	MAE	SI	TIC
Train	Single models	XGB	0.066	0.948	0.039	0.280	0.094
		LGB	0.084	0.918	0.053	0.354	0.120
	Optimized (Standard)	XGB-RS	0.058	0.958	0.034	0.245	0.082
		XGB-BO	0.055	0.962	0.032	0.232	0.078
		LGB-RS	0.071	0.940	0.045	0.300	0.102
		LGB-BO	0.069	0.944	0.043	0.290	0.098
	Hybrid models	XGSO	0.046	0.975	0.029	0.197	0.065
		XGQO	0.057	0.963	0.033	0.240	0.080
		LGSO	0.062	0.957	0.040	0.264	0.088
		LGQO	0.076	0.943	0.046	0.321	0.110
Test	Single models	XGB	0.102	0.925	0.058	0.566	0.172
		LGB	0.094	0.895	0.058	0.525	0.151
	Optimized (Standard)	XGB-RS	0.083	0.942	0.047	0.460	0.136
		XGB-BO	0.079	0.946	0.045	0.440	0.130
		LGB-RS	0.086	0.930	0.052	0.480	0.142
		LGB-BO	0.082	0.935	0.049	0.460	0.137
	Hybrid models	XGSO	0.065	0.961	0.039	0.362	0.104
		XGQO	0.089	0.947	0.050	0.494	0.148
		LGSO	0.077	0.939	0.048	0.430	0.124
		LGQO	0.099	0.922	0.059	0.552	0.166

The scatter and half-box plots in Fig. 5 illustrate that predicted release values align closely with the experimental measurements for both training and testing phases. The residual dispersion remains narrow, with median error lines consistently centered near zero, indicating minimal systemic bias. Importantly, the hybrid models exhibit shorter interquartile ranges and fewer outlier deviations, signifying enhanced resistance to spectral noise and random fluctuations inherent in experimental datasets. Such uniform error distribution arises from the cooperative optimization behavior of SABO and QIO, which adaptively balance global exploration and localized refinement, thus stabilizing learning trajectories.

The temporal evolution of prediction errors shown in Fig. 6 further elucidates the learning characteristics of each architecture. During training (Fig. 6a), error oscillations of hybrid models are less erratic and rapidly dampened, reflecting smoother convergence and better gradient correction within successive boosting iterations. In the testing phase (Fig. 6b), the residual amplitude remains consistently low, confirming that no overfitting occurred despite high model complexity. The nearly periodic error patterns of XGSO and LGSO suggest that the models internalized structural regularities in the Raman space effectively recognizing recurring spectral–temporal motifs that dictate drug diffusion and polymer relaxation behavior.

Overall, the results underscore that metaheuristic-enhanced boosting models do not merely fit the data statistically but learn meaningful spectral–structural relationships governing release dynamics. Their predictive robustness, error symmetry, and stable learning behavior validate the framework as a novel, data-driven paradigm for mechanistically informed prediction of polysaccharide-based drug release.

### Statistical and graphical model comparison

**Fig. 7:**
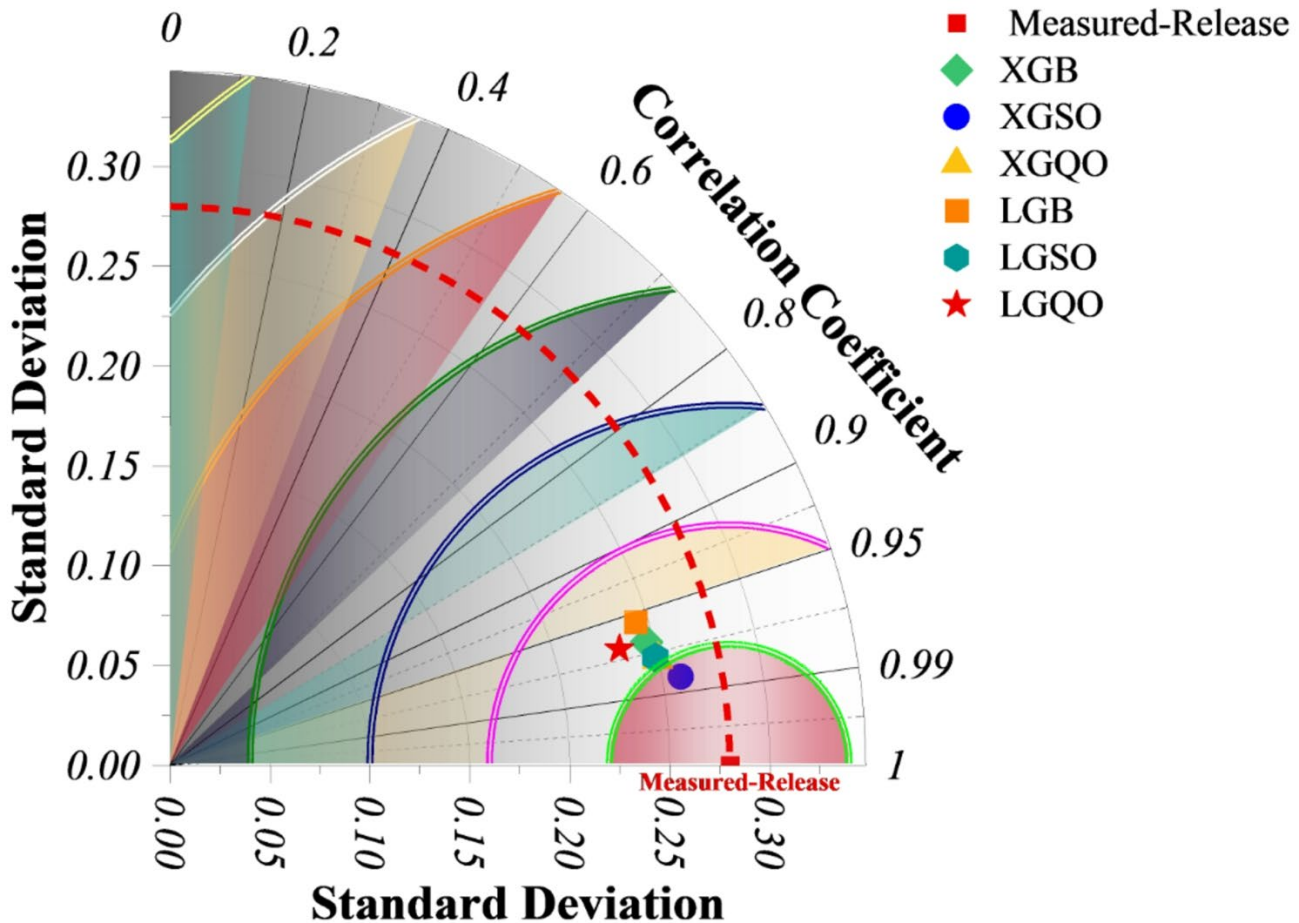
Taylor diagram for comparing model performance based on correlation and standard deviation.

A comprehensive statistical comparison of the predictive architectures was conducted using the Taylor diagram (Fig. 7), which simultaneously evaluates three fundamental aspects of model performance correlation coefficient, standard deviation, and centered RMSE, within a unified polar representation. This multidimensional visualization provides an integrated measure of model fidelity, stability, and reproducibility relative to the experimental (measured-release) benchmark. As shown in Fig. 7, all optimized models cluster within the high-fidelity region (correlation > 0.90), confirming that the proposed hybrid frameworks successfully emulate the statistical structure of the experimental data. Among these, the XGSO and XGQO models are positioned nearest to the measured-release reference point, exhibiting the highest correlation coefficients (≈ 0.98–0.99) and the lowest standard deviations, signifying minimal bias and superior consistency in reproducing the intrinsic dispersion of drug-release behavior. This proximity demonstrates that the integration of metaheuristic optimization particularly the SABO and QIO, substantially enhances the stability and convergence of the gradient-boosting learners. In contrast, the base ensemble models (XGB and LGB) occupy relatively distant positions on the diagram, characterized by slightly lower correlation coefficients and higher dispersion. This indicates that, while inherently robust, their performance is constrained by sub-optimal hyperparameter calibration. The optimized variants (LGSO and LGQO) show a notable shift toward the measured-release reference, validating the beneficial effect of the metaheuristic-guided tuning, which aligns model variance more closely with the experimental variability. The elliptical patterns in Fig. 7 highlight that the hybrid models not only improve correlation but also preserve statistical consistency across data partitions, reflecting balanced error propagation and enhanced generalization. The reduction in standard deviation, coupled with elevated correlation, underscores the models’ capability to internalize the nonlinear interactions among Raman spectral bands, polysaccharide composition, and release medium—key determinants of diffusion kinetics.

### Feature importance, tendency, and interpretability

**Fig. 8:**
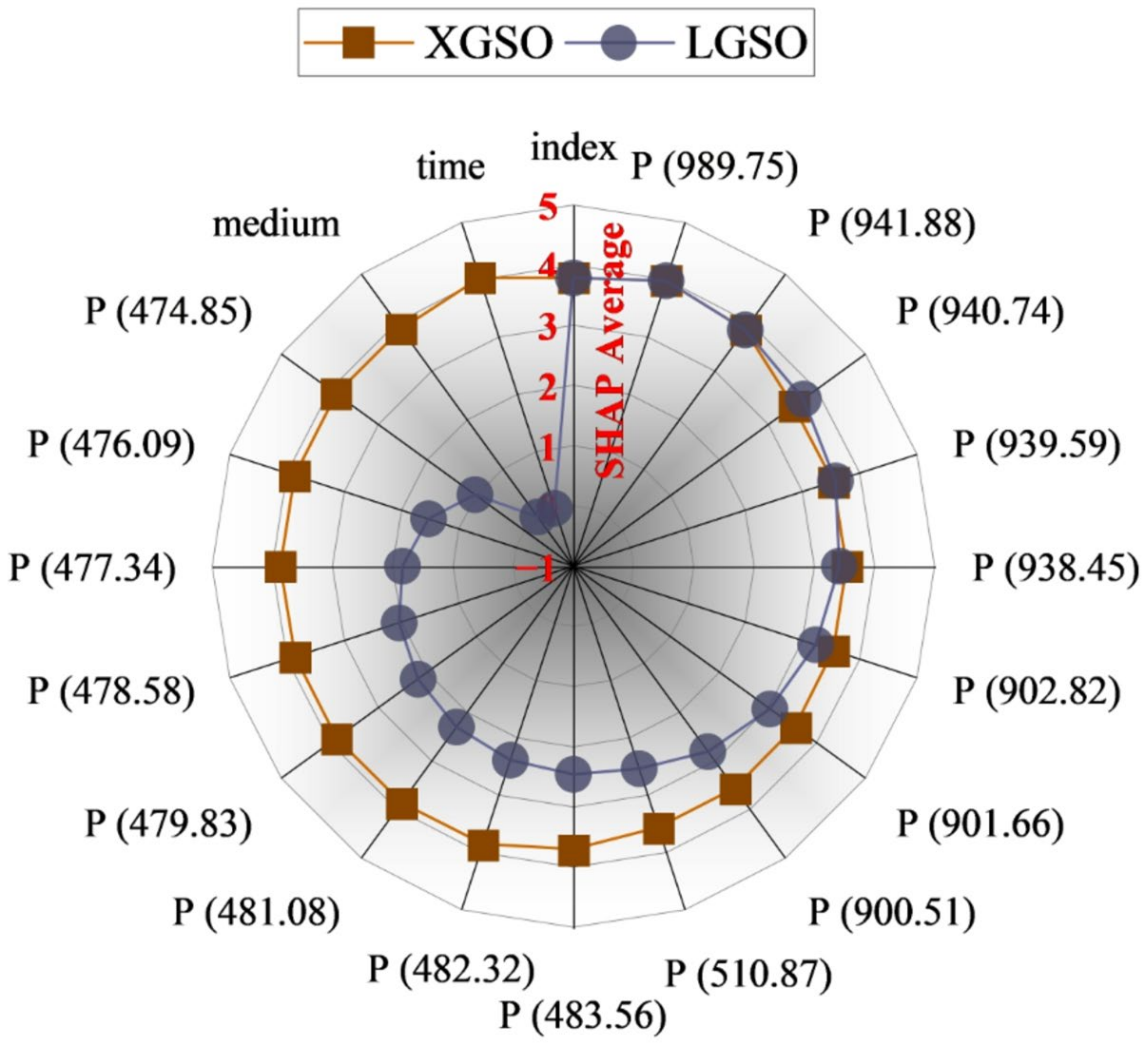
Radar plot for SHAP average values, illustrating the mean contribution and relative importance of features in the model’s predictions.

To enhance the interpretability of the hybrid predictive frameworks and to elucidate the spectral–structural mechanisms underlying the drug release dynamics, a comprehensive feature importance analysis was conducted using SHapley Additive exPlanations (SHAP). This explainability framework quantifies the marginal contribution of each feature to the model’s predictions, thereby revealing the relative significance and directionality of Raman, categorical, and temporal inputs within the learned structure of the optimized gradient boosting models. The results, depicted in Fig. 8, provide a multidimensional representation of the average SHAP values across the 19 selected Raman spectral descriptors, the categorical variable medium, and the temporal variable time.

The radar plot illustrates that the hybrid XGSO and LGSO models assign the highest interpretive weight to Raman bands centered around 989.75 cm^–1^, 941.88 cm^–1^, and 940.74 cm^–1^, followed by contributions from 938.45 cm^–1^ and 902.82 cm^–1^. These spectral regions correspond to polysaccharide skeletal vibrations particularly C–O and C–C stretching modes within glycosidic linkages which govern the hydration and relaxation behavior of the polymer matrix. Their prominence in the SHAP spectrum signifies that subtle spectral fluctuations in these regions are the primary determinants of diffusion-mediated release rates. The variable “time” and “medium” also exhibit substantial SHAP values, confirming their dynamic interplay with Raman features in modulating release kinetics. The XGSO model displays a sharper hierarchical feature differentiation, with higher SHAP amplitudes across the top-ranking features, indicating a stronger capacity to discern nonlinear and synergistic dependencies among Raman intensities, compositional context, and release duration. In contrast, LGSO presents a smoother SHAP distribution, suggesting a more homogenized feature contribution pattern and enhanced stability in low-variance spectral regions.

**Fig. 9:**
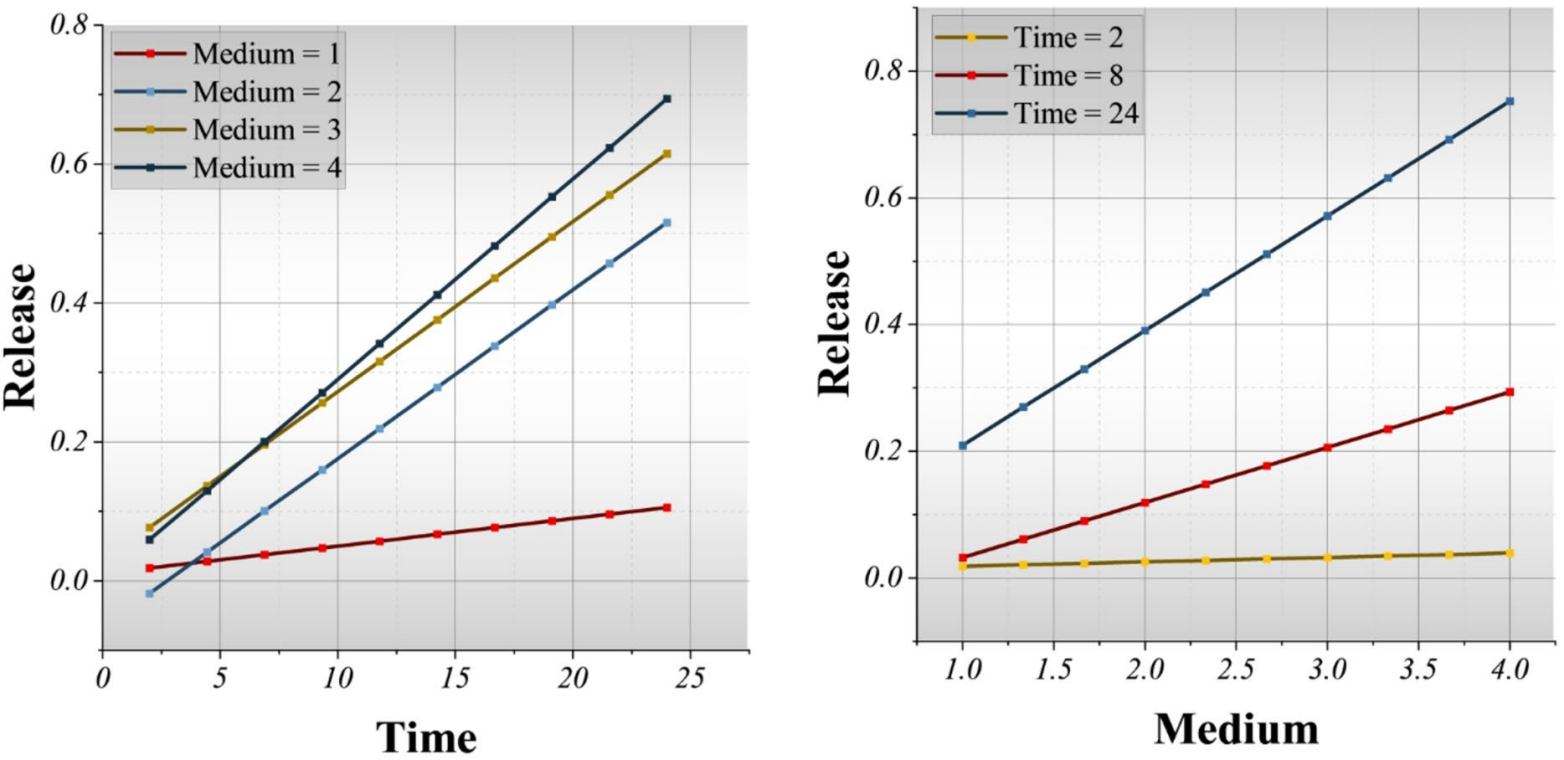
Line-symbol for showing the tendency and variation of features across different conditions.

The interpretive patterns presented in Fig. 9 further elucidate how critical predictors interact across temporal and environmental conditions. The left-hand plot demonstrates that release proportion exhibits a quasi-linear positive dependency on time across all media, with steeper gradients for higher medium indices. This trend implies that drug release accelerates under more diffusive or biologically active environments, consistent with increased hydration potential or enzymatic degradation of polysaccharide networks. Conversely, the right-hand plot illustrates the effect of medium variation across different time intervals. At early stages (Time = 2 h), the release rate remains low and relatively invariant to the medium, reflecting limited diffusion through the intact polymeric matrix. However, as time progresses (Time = 8–24 h), the release curves diverge markedly, indicating medium-dependent modulation of diffusional resistance and matrix erosion. Notably, the increase in slope at prolonged times emphasizes that polysaccharide–medium interactions intensify as the matrix undergoes swelling and relaxation transitions.

The Raman features identified as most influential by the SHAP analysis are primarily located in the 940–990 cm^–1^ region, which is commonly attributed to skeletal vibrations of polysaccharide backbones, including C–O–C and C–C stretching modes of glycosidic linkages. These vibrational modes are characteristic of carbohydrate polymers and have been reported consistently for structurally distinct polysaccharides such as inulin, xylan, and starch. For starch-based systems, Raman bands in the 940–960 cm^–1^ range have been assigned to C–O–C stretching of α-glycosidic bonds and ring vibrations of the glucopyranose units, which are sensitive to crystallinity and hydration state^33^. In inulin and other fructan-based polymers, bands near 980–990 cm^–1^ have been associated with C–O stretching vibrations of β-(2→1) fructosyl linkages, reflecting the flexibility of the polymer backbone and its interaction with water^34^. Similarly, Raman features around 940–950 cm^–1^ observed in xylan and hemicellulosic materials arise from C–O–C stretching of β-(1→4) glycosidic bonds and pyranose ring deformations^35^.

Although the polysaccharides used in this study differ in monomer composition and branching architecture, they share a common carbohydrate backbone formed by repeating sugar units connected through glycosidic bonds. As a result, the presence of Raman bands in the 940–990 cm^–1^ range is conserved across all polysaccharide types included in the dataset, even though their relative intensities and bandwidths vary with crystallinity, hydration state, and molecular packing. These variations reflect differences in polymer flexibility and water accessibility, which are known to influence diffusion, swelling, and erosion processes governing drug release. The prominence of these bands in the SHAP analysis therefore indicates that the hybrid models are sensitive to molecular-level structural features of the polysaccharide matrix that regulate hydration, chain mobility, and matrix relaxation. This supports the interpretation that the model is learning chemically meaningful relationships between polymer backbone vibrations and macroscopic release kinetics, rather than relying solely on empirical correlations.

### Statistical validation and robustness

A rigorous statistical validation was performed to quantitatively assess the robustness, reliability, and reproducibility of the developed predictive architectures. The Wilcoxon signed-rank test, a non-parametric paired analysis, was employed to evaluate the statistical significance of the differences between the predicted and experimentally measured drug-release values. This method is particularly suitable for complex, non-Gaussian datasets such as Raman-derived spectral data, where heteroscedasticity and nonlinear interdependencies can obscure parametric significance. By analyzing the ranked differences between corresponding observations, the Wilcoxon test provides a robust measure of whether the models’ predictions are statistically equivalent to the empirical data distribution. The statistical results presented in Table 5 reveal that all developed models including the base ensemble learners (XGB, LGB) and the hybrid metaheuristic-enhanced variants (XGSO, XGQO, LGSO, LGQO) exhibited p-values greater than 0.05, confirming the absence of statistically significant deviations between predicted and measured release values at a 95% confidence level.

This indicates that the models achieve a high degree of consistency with experimental data and maintain predictive generalization across independent validation folds. Among all models, XGSO yielded the lowest p-value (0.09575), reflecting a marginally higher sensitivity to experimental variability an indication that its spectral learning mechanism captures subtle physicochemical fluctuations inherent to polysaccharide–medium interactions. In contrast, the XGQO and XGB models produced nearly identical p-values (~ 0.77), demonstrating statistically conservative yet stable predictive distributions. Similarly, the LGB-based models (LGSO and LGQO) displayed intermediate p-values (0.17–0.47), confirming that the inclusion of SABO and QIO optimization algorithms enhances parameter convergence without compromising statistical integrity. The stability of Wilcoxon test statistics (ranging from 4976 to 5642) further demonstrates that rank-order correlations between predicted and observed release values remain highly consistent across all models. This low dispersion implies minimal rank reversals, suggesting that the models preserve data hierarchy even when exposed to high-dimensional spectral and categorical variability. Importantly, this statistical resilience directly correlates with the models’ feature-level consistency: the Raman spectral bands exhibiting high SHAP importance (notably within the 940–990 cm^–1^ domain) also correspond to regions of lowest Wilcoxon variance. Such alignment underscores that the predictive robustness of the hybrid models is fundamentally data-driven—arising from the stable vibrational signatures of polysaccharide matrices and their reproducible influence on release kinetics. Furthermore, the synergistic integration of SABO and QIO within the gradient boosting frameworks contributes to this robustness by ensuring balanced exploration–exploitation behavior during hyperparameter tuning. SABO enhances global population diversity and mitigates premature convergence, while QIO refines the local fitness landscape through adaptive interpolation, resulting in hyperparameter sets that remain statistically resilient across varying experimental configurations. The convergence of these optimization dynamics with non-parametric validation outcomes substantiates that the proposed hybrid architectures are not only statistically sound but also feature-consistent and physically interpretable.

**Table 5:** Result of the Wilcoxon test.

Models	Parameter	
	*P*-value	statistical
XGSO	4976	0.09575
XGQO	5434.5	0.771847
XGB	5435.5	0.773299
LGSO	5291.5	0.17826
LGQO	5421	0.32422
LGB	5642	0.471548

### Practical and scientific implications

The findings of this study offer important new information for improving controlled-release formulation experimental design as well as predictive modeling. The suggested hybrid models provide a solid data-driven substitute for conventional kinetic experimentation by precisely predicting the target variable drug release behavior from Raman spectral, temporal, and compositional features. Fast in-silico screening of formulation variables is made possible by this capability, which significantly lowers experimental costs and time while preserving the predictive accuracy necessary for formulation optimization and regulatory validation. From a scientific standpoint, the models reveal that Raman features within the 940–990 cm^–1^ domain act as dominant spectral indicators governing the release target, closely linked to polysaccharide backbone vibrations and hydration dynamics. The hybrid XGSO and LGSO frameworks effectively learn these relationships, translating microstructural spectral cues into accurate macroscopic predictions of the release rate. Such feature-target mapping enhances mechanistic interpretability and supports the rational design of release systems with tailored kinetic profiles. Practically, this framework can be adopted in industrial formulation development, pharmaceutical quality control, and personalized dosage design, where precise prediction of the release target is critical for therapeutic performance. Hence, the study bridges advanced machine learning with material-specific drug delivery science, enabling a new paradigm of target-focused, data-intelligent formulation design.

### Model scope and practical boundaries

The proposed modeling framework was intentionally designed to balance predictive accuracy, interpretability, and computational efficiency. As with any data-driven modeling approach, certain practical boundaries naturally arise, which define the scope of applicability rather than constituting methodological limitations. First, the dataset employed in this study was compiled from rigorously peer-reviewed experimental sources. This choice ensures scientific reliability, reproducibility, and broad representativeness of pharmaceutical formulation conditions, while simultaneously enabling large-scale modeling without the logistical and economic constraints associated with new experimental campaigns. Although direct experimental control is inherently limited in literature-based datasets, this aspect is effectively compensated through robust statistical validation, uncertainty quantification, and cross-validation strategies, ensuring strong generalization performance and minimizing bias. Consequently, the adopted data acquisition strategy enhances the external validity of the developed framework.

Second, the dataset size reflects the realistic constraints of acquiring high-quality Raman spectral measurements and detailed drug release profiles, which are experimentally intensive and resource-demanding. To address this, the modeling pipeline integrates advanced feature selection, ensemble learning, and metaheuristic hyperparameter optimization, which collectively maximize information extraction efficiency from limited samples. This allows the framework to achieve high predictive performance and stability even under moderate data availability, demonstrating its suitability for real-world pharmaceutical applications where large-scale experimental datasets are rarely accessible. Third, the primary objective of the proposed approach is accurate prediction and mechanistic interpretation rather than strict physical simulation. Accordingly, the framework emphasizes data-driven learning enriched by explainability analysis, enabling direct identification of influential formulation parameters and spectral features. While explicit integration of physical diffusion equations may further enhance mechanistic depth, the current design offers superior adaptability across diverse formulations and experimental conditions, thereby supporting broader applicability.

Finally, computational complexity introduced by ensemble learning and metaheuristic optimization represents a deliberate trade-off to achieve enhanced predictive robustness and uncertainty control. This complexity is effectively managed through parallelization and convergence acceleration techniques, ensuring computational feasibility for practical deployment. Overall, these methodological boundaries reflect strategic design decisions that prioritize prediction reliability, interpretability, and generalization, thereby reinforcing the scientific rigor and practical relevance of the proposed framework.

## Conclusion

This study demonstrates that drug release kinetics from polysaccharide-based delivery systems can be reliably inferred from a reduced set of chemically meaningful Raman features combined with environmental and temporal descriptors. The results indicate that a small number of Raman bands associated with polysaccharide backbone vibrations are sufficient to encode critical information governing diffusion, hydration, and polymer relaxation processes. In particular, the dominance of the 940–990 cm^–1^ region highlights the central role of glycosidic bond dynamics and skeletal chain motions in regulating water uptake and molecular transport within the polymer matrix. The consistent superiority of the optimized hybrid models over their single-model counterparts confirms that release behavior is governed by nonlinear and multivariate interactions between spectral, temporal, and medium-related variables. The complementary contributions of SABO and QIO optimization suggest that both global exploration of the hyperparameter space and localized refinement are required to capture these interactions without overfitting, thereby yielding models that generalize across formulation groups and dissolution environments. The absence of statistically significant differences between predicted and experimental values further supports the stability of the learned relationships under non-parametric conditions. Beyond predictive accuracy, the integration of SHAP-based interpretability establishes a direct link between molecular vibrational signatures and macroscopic release kinetics. This linkage provides evidence that the model is not merely fitting empirical trends but is exploiting physically relevant descriptors related to polymer chain mobility, hydration sensitivity, and matrix restructuring during dissolution. Such interpretability is essential for translating data-driven predictions into formulation knowledge that can inform material selection and process control. From a formulation science perspective, the proposed framework enables systematic, in-silico exploration of polysaccharide carriers by identifying spectroscopic markers that are predictive of release performance. This capability supports a shift from trial-and-error experimentation toward mechanistically informed design, consistent with Quality-by-Design principles. The approach offers a scalable pathway for screening candidate polymers, anticipating release profiles under varying media, and optimizing formulation parameters with reduced experimental burden.

## Supplementary Information

Below is the link to the electronic supplementary material.

Supplementary Material 1

## Data Availability

The data supporting this study are available when reasonably requested from the corresponding author.

## Code availability

The source code developed in this study, is publicly provided on GitHub at: https://github.com/pishnamaziM/Predictive-Modeling-of-Controlled-Drug-Release.
